# Lrp4 Domains Differentially Regulate Limb/Brain Development and Synaptic Plasticity

**DOI:** 10.1371/journal.pone.0116701

**Published:** 2015-02-17

**Authors:** Theresa Pohlkamp, Murat Durakoglugil, Courtney Lane-Donovan, Xunde Xian, Eric B. Johnson, Robert E. Hammer, Joachim Herz

**Affiliations:** 1 Department of Molecular Genetics, University of Texas Southwestern Medical Center, Dallas, Texas, 75390, United States of America; 2 Department of Biochemistry, University of Texas Southwestern Medical Center, Dallas, Texas, 75390, United States of America; 3 Department of Neuroscience, University of Texas Southwestern Medical Center, Dallas, Texas, 75390, United States of America; 4 Department of Neurology and Neurotherapeutics, University of Texas Southwestern Medical Center, Dallas, Texas, 75390, United States of America; 5 Center for Translational Neurodegeneration Research, University of Texas Southwestern Medical Center, Dallas, Texas, 75390, United States of America; University of Sydney, AUSTRALIA

## Abstract

Apolipoprotein E (ApoE) genotype is the strongest predictor of Alzheimer’s Disease (AD) risk. ApoE is a cholesterol transport protein that binds to members of the Low-Density Lipoprotein (LDL) Receptor family, which includes LDL Receptor Related Protein 4 (Lrp4). Lrp4, together with one of its ligands Agrin and its co-receptors Muscle Specific Kinase (MuSK) and Amyloid Precursor Protein (APP), regulates neuromuscular junction (NMJ) formation. All four proteins are also expressed in the adult brain, and APP, MuSK, and Agrin are required for normal synapse function in the CNS. Here, we show that Lrp4 is also required for normal hippocampal plasticity. In contrast to the closely related Lrp8/Apoer2, the intracellular domain of Lrp4 does not appear to be necessary for normal expression and maintenance of long-term potentiation at central synapses or for the formation and maintenance of peripheral NMJs. However, it does play a role in limb development.

## Introduction

ApoE receptors are essential regulators of excitatory innervation of central and neuromuscular synapses. In the brain, the ε4 isoform of ApoE (ApoE4) impairs ApoE receptor recycling, thereby inducing synaptic dysfunction and sensitizing the synapse to amyloid-β (Aβ) mediated functional suppression [1]. The Aβ peptide is the ultimate proteolytic processing product of APP and, as the amyloid hypothesis posits, essential at least for the initiation of AD pathogenesis [2]. Synaptic dysfunction induced by Aβ can be prevented at early stages of amyloid accumulation by Reelin-mediated activation of Lrp8/Apoer2 [3].

Reelin, like the Lrp4 ligand Agrin, is a large, structurally complex signaling molecule that binds to the LDL Receptor family members VLDLR and Lrp8/Apoer2. Reelin and its receptors are essential for brain development and neuronal positioning [4]. In the adult brain, Reelin is mainly expressed by a subset of interneurons and layer V pyramidal neurons [5] and, together with its receptors, is essential for the regulation of plasticity at excitatory synapses [6,7].

Intriguingly, at central and peripheral synapses APP is part of an organizing center around which ApoE receptors and their ligands assemble through mutual interaction, resulting in potentiation of Reelin and Agrin mediated signal transduction, respectively [7–10]. Moreover, several ApoE receptors, including Lrp1, Apoer2, and Lrp4 are highly expressed in muscle tissues [10], and ApoE genotype influences neuromuscular disease of various etiology in a similar way as in AD [11], suggesting that ApoE-ApoE receptor interactions could also affect muscle innervation.

In the muscle, Lrp4 serves as a receptor for Agrin and is essential for the activation of MuSK, the clustering of acetylcholine receptors, and thus the formation of the NMJ as a whole [10,12–14]. Genetic disruption of Lrp4, MuSK, or Agrin abrogates NMJ formation, and loss of APP together with its closely related family member APLP2 severely impairs it. In contrast to Reelin, which is critical for brain development and also regulates synaptic plasticity, Agrin does not appear to be important for the structural formation of the CNS [15]. By contrast, the same proteins that interact in the periphery with Lrp4 to form and maintain the NMJ—Agrin, MuSK, and APP—are also required for normal plasticity at central synapses [2,16–18]. On the other hand, Lrp4, but not Agrin, MuSK or APP, is essential for limb development, where Lrp4 functions as a modulator of Wnt signaling in the apical ectodermal ridge [19]. This kind of multifunctionality is common for LDL receptor family members [4,20].

Taken together, these findings suggest that ApoE receptors and their interacting partners are pivotal parts of a fundamental synaptic homeostatic mechanism. Genetic predispositions that impair this mechanism in favor of the synapse-suppressing Aβ, such as ApoE4 genotype and mutations in APP or the presenilins, which release Aβ from APP, lead to an imbalance of Aβ levels and initiate AD pathogenesis. Lrp4 is expressed in the adult brain [21] and thus may participate in synapse function there as well.

Here, we show a comprehensive phenotypic analysis of an allelic series of Lrp4 knock-in mice to determine the physiological functions of various motifs contained within the Lrp4 intracellular domain (ICD). Limb development emerged as a sensitive indicator, which allowed us to discern functional requirements for the ICD during the embryonic stage. We also investigated the functional role of the Lrp4 cytoplasmic domain in the brain. Considering the high expression of Lrp4 in the central and peripheral nervous system, we investigated potential roles for Lrp4 in brain development. Finally, since Lrp4 is required for the function of NMJs, and its partners MuSK, Agrin, and APP have well-documented roles at central synapses, we investigated the role of Lrp4 in the function of central excitatory synapses, including whether the ICD of Lrp4 is mechanistically equally important as the Lrp8/ApoER2-ICD for synaptic function [7].

## Materials and Methods

### Ethics statement

All animal care protocols were followed and experimental procedures were performed in accordance with and approved by the Institutional Animal Care and Use Committee (IACUC) of the University of Texas Southwestern Medical Center at Dallas. Mice were deeply anesthetized with isoflurane and sacrificed.

### *Lrp4* KO and KI mice

*Lrp4* ^*-/-*^ mice, in which the first exon is deleted, and *Lrp4* ^*ECD/ECD*^ KI mice have been described before [10,22]. Briefly, to generate *Lrp4* ^*ECD/ECD*^, a stop codon was inserted into *Lrp4* exon 36 followed by a bovine growth hormone 3’-UTR. The 1905 amino acid Lrp4 is truncated at residue 1722, by mutating the triplet CAC encoding His1723 to TAA. *Lrp4* ^*ECD/ECD*^ KI mice lack the membrane spanning domain (residues 1726–1746) and the intracellular domain (residues 1747–1905) of Lrp4. To generate the other KI mice of the allelic series, similar constructs were generated [22]: Briefly, *Lrp4* ^*WT-KI/WT-KI*^ encodes the full-length Lrp4 protein, but lacking introns 36 and 37, which were replaced by a cDNA expression cassette. In *Lrp4* ^*AS/AS*^, a naturally occurring splice variant was generated as described [22]. The NPSY-motif (residues 1776–1779) was mutated to AAAA to generate *Lrp4* ^*AAAA/AAAA*^; the PDZ-domain binding motif (residues 1902–1905) was truncated by insertion of a stop codon (TAG) following Ser1901 to generate *Lrp4* ^*ΔPDZ/ΔPDZ*^; *Lrp4* ^*LDLR-ICD/LDLR-ICD*^ replaces the Lrp4-tail downstream of Arg1747 with the ICD of LDLR starting at Asp814; and a myc-epitope was inserted following Lys1753 to generate *Lrp4* ^*ΔICD/ΔICD*^. Finally, all constructs of the allelic sequence contain the bovine growth hormone 3’UTR [22]. Residue numbering is based on UniProtKB identifier Q8V156 for Lrp4 and P35951 for LDLR.

### Perfusion, Sectioning and Immunohistochemistry

Perfusion-fixed brains (4% PFA in PBS) of adult mice (2–3 months) were embedded in 5% agarose (in PBS) and 50 μm sections were cut with a Leica VT 1000S Vibratome. Sections were blocked for 1h in PBS with 0.05% Triton X (PBST) containing 10% donkey serum. Primary antibodies mouse anti-NeuN (1:1000, Millipore), rabbit anti-Brn1 (1:1000, Santa Cruz), were diluted in blocking medium and slices were incubated overnight at 4°C. After washing with PBST, the slices were incubated with secondary AlexaFluor (AF594 or 499)-coupled antibodies (donkey, Invitrogen) for 2h at room temperature. Slices were washed once with PBST containing DAPI (1:10.000), then twice with PBST, and mounted using Permanent Mounting Medium (VectaMount, Vector).

### Alizarin Red/Alcian Blue Staining

Mice were sacrificed, limbs were removed and pictures were taken with a Nikon digital camera at 0.63X magnification. Limbs were then skinned and placed in PBS to remove as much soft tissue as possible. The skeleton was fixed in 95% ethanol for five days, briefly rinsed with water, and then transferred to acetone for two days to remove remaining fat. After rinsing the samples with water, they were incubated in the staining solution (0.015% alcian blue (Sigma #A-5268), 0.005% alizarin red (Sigma #A-5533), 5% glacial acetic acid, 68% ethanol) at 37°C for three days. Skeletons were briefly rinsed with water and transferred to 1% KOH for three days. Samples were further cleared by passage through a glycerol gradient: 20% to 50% to 80% glycerol with 1% KOH, each for five days. Prior to taking the pictures, the skeletons were transferred into 100% glycerol.

### Extracellular field recordings

Hippocampal slices were prepared from 2–4 month old *Lrp4* ^*WT-KI/WT-KI*^, *Lrp4* ^*ECD/ECD*^, and *Lrp4* ^*ΔICD/ΔICD*^ mice. The brain was quickly removed and placed in an ice-cold high-sucrose cutting solution containing 110 mM sucrose, 60 mM NaCl, 3 mM KCl, 1.25 mM NaH_2_PO_4_, 28 mM NaHCO_3_, 0.5 mM CaCl_2_, 5 mM glucose, 0.6 mM ascorbic acid, and 7 mM MgSO_4_. 400 μm transverse sections were cut using a vibratome. Slices were then placed into the incubation chamber containing 50% artificial cerebrospinal fluid (aCSF; 124 mM NaCl, 3 mM KCl, 1.25 mM NaH_2_PO_4_, 26 mM NaHCO_3_, 10 mM D-glucose, 2 mM CaCl_2_, 1 mM MgSO_4_) and 50% sucrose-containing solution. After 1 hour of recovery, slices were transferred to an interface recording chamber, kept at 31°C and perfused with aCSF with a flow rate of 2–3 ml/min. For stimulation, concentric bipolar electrodes were used (FHC, Catalog no CBBRC75, 1201 Main St, Bowdoin, ME 04287, USA), and placed into the Stratum radiatum. Stimulus intensity was set at 40–60% of maximum response (calculated before acquisition of baseline) and delivered thorough an Isolated Pulse Stimulator (A-M Systems, Model 2100). A custom written program in Labview 7.0 was used for recording and analysis of LTP experiments (courtesy of Dr. Jay Gibson, UT Southwestern). A theta-burst (TBS; train of 4 pulses at 100-Hz repeated 10 times with 200 ms intervals; repeated 5 times at 10 s intervals) was used as the conditioning stimulus.

## Results and Discussion

**Fig 1:**
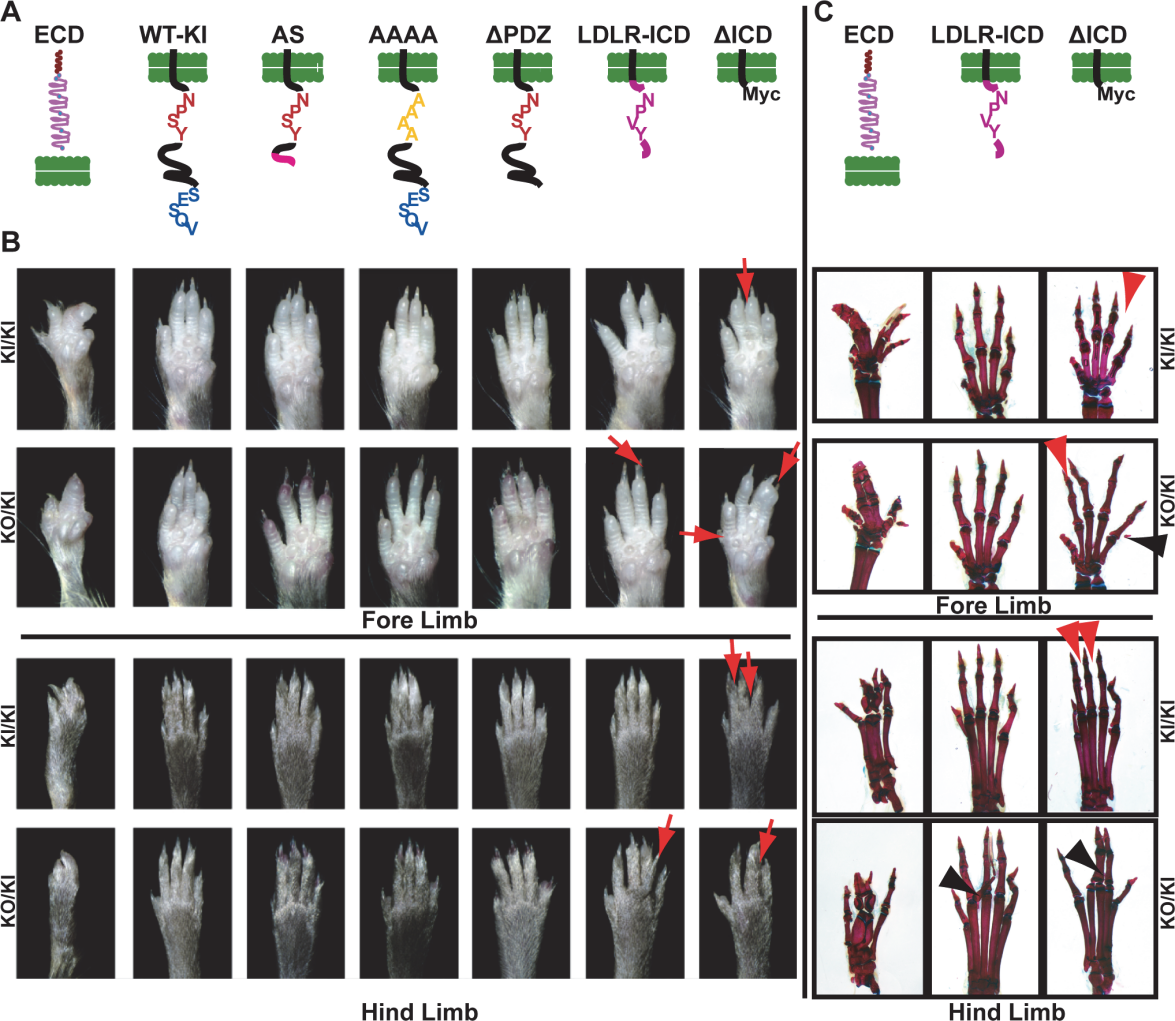
Limb and bone structure of different *Lrp4* KI mutants. **A**: Illustration of the different Lrp4 protein products of all KI mutants. Panels are aligned to paw images in B and C to indicate genotypes. **B**: Ventral view of fore and hind limbs of *Lrp4* KI mutants. Homozygous mutant mice for each allelic variant (*KI/KI*) and compound mutant mice that carry one allelic variant and one KO allele (*KO/KI*) are shown. Note that there are strong defects in the limb pattering of *Lrp4* ^*ECD/ECD*^, intermediate defects in *Lrp4* ^*ΔICD/ΔIC*^, and only mild defects in *Lrp4* ^*LDLR-ICD/LDLR-ICD*^ (red arrows). **C**: Ventral view of alizarin red (stains bones) and alcian blue (stains cartilage) of different *Lrp4* KI mutants. A WT-KI allele (2^nd^ panel in A and B) was generated to control for the lack of introns in the ICD-cassette in the other KI mutants. Black arrowheads: ectopic bone or bony fusion; red arrowheads: soft-tissue fusion. (modified from [22]).

*Lrp4* mutations cause mulefoot disease in cattle [23,24] and Cenani-Lenz syndrome in humans, a developmental disorder with limb and kidney defects [25] that are recapitulated in *Lrp4* mutant mice [4,19,23,26]. Lrp4 shares a similar structure with other members of the LDLR family. It has a large extracellular ligand-binding domain, a transmembrane domain, and a short cytoplasmic tail that contains an NPxY and a PDZ-binding motif [27]. To study the functional importance of these motifs in vivo, we generated an allelic series of *Lrp4* knock-in lines (KIs) [22]. These mice include: (1) *Lrp4* ^*ECD/ECD*^, in which the ICD and transmembrane domain are deleted, resulting in a secreted extracellular domain (ECD) [10,19] (2) *Lrp4* ^*WT-KI/WT-KI*^, in which a cDNA encoding the wild type ICD was knocked-in as a control; (3) *Lrp4* ^*AS/AS*^, containing an alternatively spliced (AS) form of *Lrp4* that truncates the ICD after the NPxY motif; (4) *Lrp4* ^*AAAA/AAAA*^, in which the NPxY motif is mutated to AAAA; (5) *Lrp4* ^*ΔPDZ/ΔPDZ*^, lacking the C-terminal PDZ-interaction motif; (6) *Lrp4* ^*LDLR-ICD/LDLR-ICD*^, in which the Lrp4-ICD is replaced with the LDLR-ICD; and (7) *Lrp4* ^*ΔICD/ΔICD*^, in which the ICD is replaced by a Myc-tag [10] and models an Lrp4 truncation found in cattle suffering from mulefoot disease [23] (Fig. 1A).

Surprisingly, limb development was unaffected in most of the ICD KI mice, with only a mild polysyndactyly phenotype in the *Lrp4* ^*LDLR-ICD/LDLR-ICD*^ and *Lrp4* ^*ΔICD/ΔICD*^ lines (Fig. 1B,C). The phenotype of the Lrp4^*LDLR-ICD/LDLR-ICD*^ and *Lrp4* ^*ΔICD/ΔICD*^ lines was further enhanced when one mutant *Lrp4* allele was replaced by a true *Lrp4* null allele [26]. Conversely, polysyndactyly is fully penetrant in *Lrp4* ^*ECD/ECD*^ mice (Fig. 1B,C). These results indicate that membrane anchoring of Lrp4 is important for limb development, while sequence motifs in the ICD are of more limited functional importance [28].

**Fig 2:**
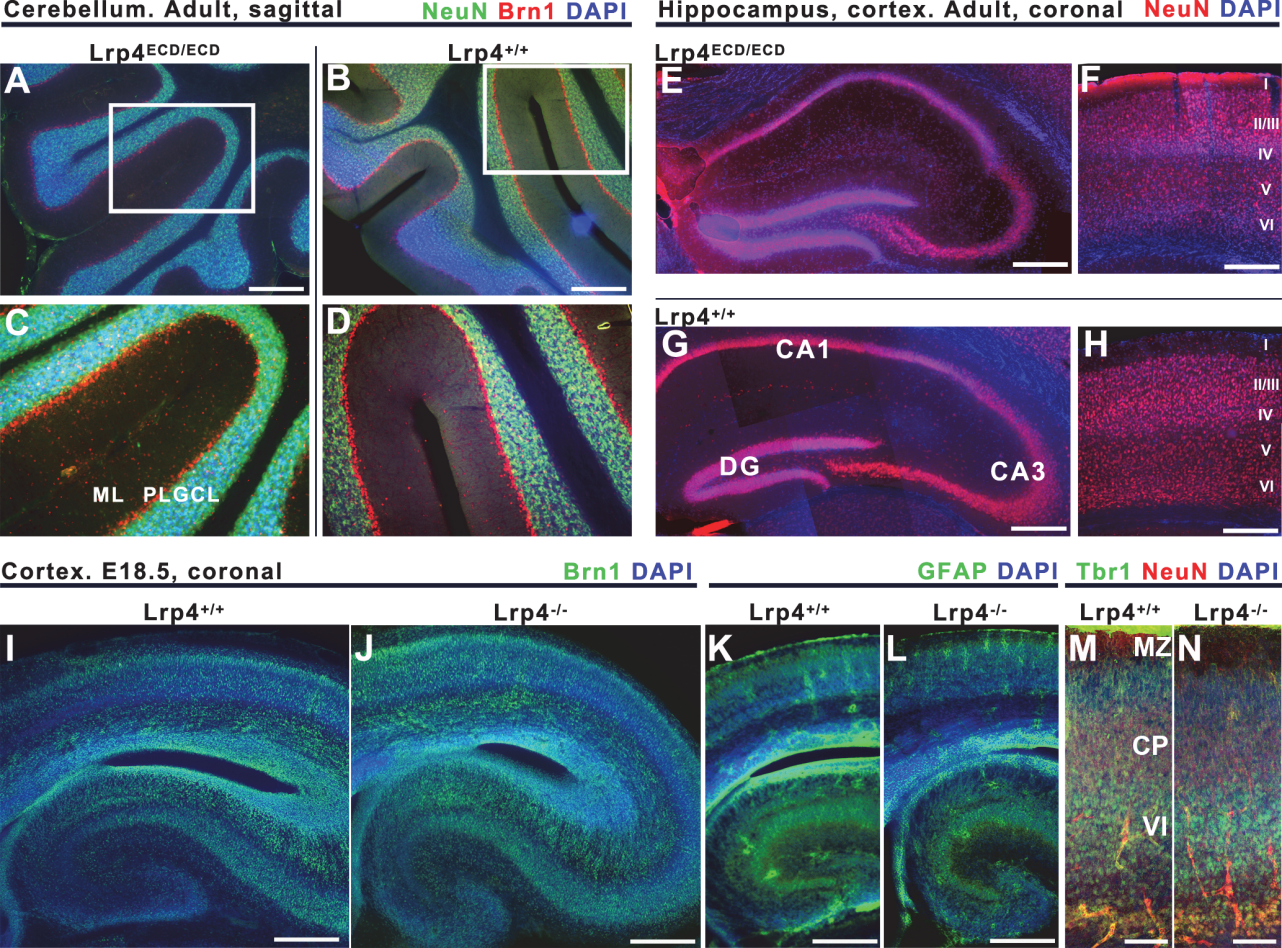
Normal brain development in *Lrp4* ^*ECD/ECD*^ and *Lrp4* ^*-/-*^ mice. **A-D**: Sagittal slices of the *Lrp4* ^*ECD/ECD*^ (A,C) and wild type (*Lrp4* ^*+/+*^ B,D) mouse cerebellum labeled with NeuN (green), Brn1 (red) and DAPI (blue). Brn1 and NeuN are commonly used markers to label neurons. ML = molecular layer, PL = Purkinje cell layer, and GCL = granule cell layer are clearly distinguishable and not different in the cerebellum of *Lrp4* ^*ECD/ECD*^ and *Lrp4* ^*+/+*^ adult mice (>2 months). **E-H**: Coronal sections of *Lrp4* ^*ECD/ECD*^ (E,F) and *Lrp4* ^*+/+*^ (G,H) brains showing hippocampus (E,G) and somatosensory cortex (F,H). Slices are labeled for NeuN and DAPI to visualize normal cortical lamination (layers I-VI). I-N: Coronal sections of E18.5 *Lrp4* ^*-/-*^ brains compared to their wild type litter mates. Brn1 (I,J) and GFAP (K,L) immunoreactivity in the cortex and hippocampus and Tbr1 plus NeuN double labeling in the cortex are illustrated. Scale bars = 200 μm (A-H), 400 μm (I-L), 100 μm (M,N).

Lrp4 is also required for NMJ formation [10,12–14]. *Lrp4* knock-out (KO) mice die perinatally due to respiratory failure from a complete lack of functional NMJs [13]. *Lrp4* mRNA is expressed in CNS neurons, particularly the hippocampus, suggesting a role for Lrp4 in CNS synapse function [21]. We first examined brain development in *Lrp4* ^*ECD/ECD*^ mice, since they had the strongest limb phenotype that was compatible with perinatal survival [10,19,23]. Sections of adult brains (n = 4, age range 2–5 months) were stained with the neuronal markers NeuN and Brn1, and DAPI. No gross abnormalities were observed in the *Lrp4* ^*ECD/ECD*^ brain in the cerebellum (Fig. 2A-D), hippocampus (Fig. 2E,G) or cortex (Fig. 2F,H). These results suggest that Lrp4 is not required for CNS development, in stark contrast to other members of the LDLR family, including Lrp2/Megalin, Vldlr, and Lrp8/Apoer2 [29–31]. While this manuscript was under review a related article was published showing that transgenic *Lrp4* ^*-/-*^ mice, in which lethality was rescued by muscle-specific Lrp4 expression (*Lrp4* ^*-/-*^ *;Lrp4* ^*m*^) have normal brain anatomy[32]. This concurs with the normal pattern of neurons observed in the adult brain of *Lrp4* ^*ECD/ECD*^ mice. Moreover, we noticed no abnormalities in sections stained for Map2 (dendrites), Ctip2 (layer V), Tbr1 (layer VI neurons), and GFAP, nor could we find anatomical differences in prenatal *Lrp4* ^*-/-*^ brains (Fig. 2I-N).

**Fig 3:**
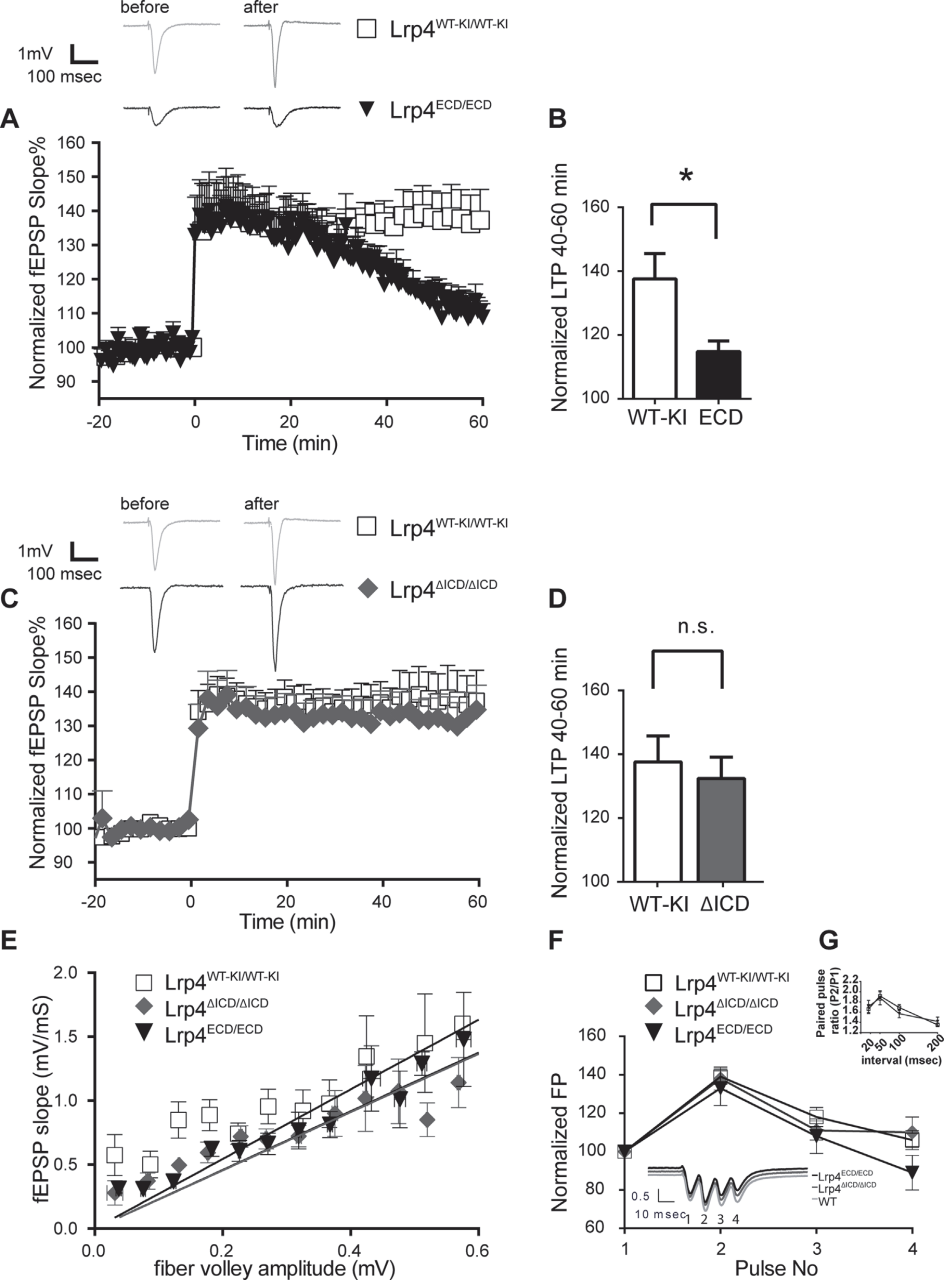
LTP is impaired in *Lrp4* ^*ECD/ECD*^ but not in *Lrp4* ^*ΔICD/ΔICD*^ mice. **A**: *Upper Panel*, Sample traces before and 40 min after theta-burst stimulation (TBS); *Lower Panel*, Results of experiments from *Lrp4* ^*ECD/ECD*^ mice compared to their *Lrp4* ^*WT-KI/WT-KI*^ controls. TBS induced on average a 37.63 ± 7.88% increase in *Lrp4* ^*WT-KI/WT-KI*^ control slices (open squares, n = 16, N = 5), but only 14.83 ± 3.39% LTP in slices from the *Lrp4* ^*ECD/ECD*^ mice (black triangles, n = 10, N = 3). **B**: Unpaired t-test was used to compare each sample for LTP calculated 40–60 min after theta-burst. Values are the means of the normalized fEPSP slopes. * denotes significance, p = 0.0387. **C:** *Upper Panel*, Sample traces before and 40 min after TBS. *Lower Panel*, Results of experiments from *Lrp4* ^*ΔICD/ΔICD*^ mice compared to their *Lrp4* ^*WT-KI/WT-KI*^ controls. *Lrp4* ^*WT-KI/WT-KI*^ slices were recorded on consecutive days and used as internal controls and pooled together. TBS induced a 37.63 ± 7.88% LTP in *Lrp4* ^*WT-KI/WT-KI*^ control slices (open squares, n = 16, N = 5), and 32.42 ± 6.27% LTP in slices from the *Lrp4* ^*ΔICD/ΔICD*^ mice (gray filled rhombus, n = 11, N = 5). **D**: There was no significant difference in LTP between *Lrp4* ^*WT-KI/WT-KI*^ *and Lrp4* ^*ΔICD/ΔICD*^ mice (p = 0.63). **E.** Input-output curves calculated as a function of fiber volley amplitude to the slopes of fEPSP’s. Average peak amplitudes for *Lrp4* ^*WT-KI/WT-KI*^, *Lrp4* ^*ECD/ECD*^ and *Lrp4* ^*ΔICD/ΔICD*^ slices used in the experiments were 1.68 ± 0.15 mV, 1.27 ± 0.25 mV, and 1.50 ± 0.20 mV), respectively, and were not significantly different from each other. (One-way ANOVA, F = 1.124, p = 0.33.) **F:** Theta-burst analysis or **G:** paired pulse ratios (n = 8, N = 3 for each) did not reveal any significant differences between *Lrp4* ^*WT-KI/WT-KI*^ and *Lrp4* ^*ECD/ECD*^ (two-way ANOVA, F(3,56) = 0.46, p = 0.71). N = number of animals.

The essential function of Lrp4 in the peripheral nervous system has precluded the evaluation of the consequences of a complete loss of Lrp4 in the adult mouse brain. However, the hypomorphic strains we have described here are fully viable, which has allowed us to study the effect of altered or reduced Lrp4 signaling on synaptic function and plasticity in the adult brain. *Lrp4* mRNA is strongly expressed in the granule cell layer of the dentate gyrus and the pyramidal cell layer of the cornus ammonis (CA) of the hippocampus, a region known to be required for learning and memory [21]. Additionally, Lrp4 is enriched in the post-synaptic density (PSD) fraction of mouse forebrain and its ICD has been shown to interact with the synaptic scaffolding protein PSD-95 [21,23] and other synaptic, PDZ-domain containing proteins [23] that are directly or indirectly involved in NMDA and/or AMPA receptor function (including LIN-7B/MALS-2 [33], ARIP2 [34–36], GIPC1/Synectin [37], Erbin/ERBB2IP/SERBIN [38,39]). NOS1AP (nNOS adaptor protein) binds to the Lrp4 NPXY-motif [23], and this is another mechanism by which Lrp4 may affect NMDA receptor function [40]. Taken together, these interactions suggest several possible functional interactions by which the Lrp4-ICD can affect glutamate receptors to affect synaptic plasticity. We therefore examined synaptic function in the *Lrp4* ^*ΔICD/ΔICD*^ and *Lrp4* ^*ECD/ECD*^ mice using extracellular field recordings of theta-burst long-term potentiation (LTP) in CA3-CA1 Schaeffer collaterals. LTP is an activity-dependent, enduring increase in the strength of synaptic connections and therefore an important mechanism for learning and memory formation. CA3-CA1 projections are a well-studied model for understanding and measuring synaptic plasticity. Lrp4 expression is elevated in the CA1 region of the hippocampus, which receives these CA3 projections [21]. Whereas *Lrp4* ^*ECD/ECD*^ mice show a strong deficit in late-phase LTP compared to *Lrp4* ^*WT-KI/WT-KI*^ controls (Fig. 3A,B) *Lrp4* ^*ΔICD/ΔICD*^ mice have no deficits in LTP (Fig. 3C,D). Neither genotype exhibited a change in baseline neurotransmission, as shown by input-output curves, (Fig. 3E), or a change in short-term synaptic plasticity, as evidenced by the lack of a deficit in paired-pulse facilitation (Fig. 3F). Finally, since we did not observe changes in LTP in mice lacking only the Lrp4-ICD, we suspect that the interactions of the Lrp4-ICD with the resident synaptic proteins detailed above are not critical for the effect of Lrp4 on synaptic plasticity at the level we have examined. An alternative explanation would be that the function of the Lrp4-ICD is compensated for by other lipoprotein receptors or by other membrane proteins at the synapse. Moreover, while the Lrp4-ECD sufficiently supports NMJ development to allow postnatal survival, it is nevertheless dysfunctional in the adult brain. This is consistent with the findings by Gomez et al. [32], who showed that mice lacking Lrp4 entirely in their brains also had normal neuroanatomy, but impaired electrophysiology.

We have shown that signal transduction by Lrp4 through the ICD is not required for synaptic plasticity, which contrasts with the requirement of the Lrp8/Apoer2 ICD [7]. The interaction between PSD-95 and Lrp4 requires the PDZ binding motif at the Lrp4 C-terminus [23], and replacement of the entire ICD with a Myc tag (*Lrp4* ^*ΔICD*^) [10] did not affect synaptic plasticity in our experiments (Fig. 3C,D). This could indicate that an interaction between Lrp4 and the PSD scaffold that also recruits NMDA receptors into a large complex is not essential for synaptic plasticity, because Lrp4 is retained in the complex by extracellular domain interactions with other postsynaptic proteins, such as APP [10]. The absence of plasticity deficits in *Lrp4* ^*ΔICD/ΔICD*^ mice coincides with normal NMJ development and function [10,41]. The prominent LTP defect in the *Lrp4* ^*ECD/ECD*^ brain slices suggest that, as in the NMJ, the loss of the membrane anchor severely impairs Lrp4 function, or that secretion of the soluble Lrp4 ectodomain acts in a suppressive manner on other receptors or components of the PSD, such as APP or glutamate receptors, or by scavenging Agrin [10].

At the NMJ, Agrin binds and clusters Lrp4 and MuSK, enhancing MuSK activation [12]. MuSK and Agrin are both expressed in the brain, and inhibitory avoidance learning increases the mRNA and protein levels of each in the hippocampus [17]. Moreover, knockdown of MuSK prevents memory consolidation [17]. *Lrp4* ^*ECD/ECD*^ mice have an LTP deficit similar to that observed in a MuSK knockdown, suggesting that both receptors functionally collaborate in central synapses as they do in the NMJ. Thus, in the brain as at the NMJ, the Lrp4 ECD might bind Agrin and interact with MuSK [42]. Consequently, the contribution of Lrp4 to the interaction and clustering of receptors and ligands at the central synapse, in cis and/or trans, may be its primary function and dependent on membrane anchoring, whereas the function of its ICD might be minor or compensated for by other (lipoprotein) receptors in the cluster.

Furthermore, APP interacts with Lrp4 to augment NMJ formation [10]. Intriguingly, APP is the precursor to Aβ, the main toxic particle of AD. APP and Aβ both play significant roles in the brain function and are known to interact with other members of the LDLR family at central synapses [4]. Lrp4 may also interact with APP in the CNS to affect disease processes, and this interaction should be explored. Finally, ApoE4, the main genetic risk factor for AD and a ligand for Lrp4, accelerates synaptic dysfunction, and the role of Lrp4 in this process has yet to be determined.

In summary, we have examined the *in vivo* roles of the ICD and ECD of Lrp4 in limb development and CNS function. Our findings show that while Lrp4 does not seem to play a major role in the development of the CNS, it does modulate synaptic plasticity. The hypomorphic phenotype of the *Lrp4* ^*ECD/ECD*^ mice will be useful to test whether ApoE4 genotype further confounds their synaptic deficit, suggesting another possible role for the ApoE receptor Lrp4 in early synaptic dysfunction in Alzheimer’s disease.
